# Electroacupuncture relieves hyperalgesia by regulating neuronal–glial interaction and glutamate transporters of spinal dorsal horns in rats with acute incisional neck pain

**DOI:** 10.3389/fnins.2022.885107

**Published:** 2022-10-26

**Authors:** Jun-ying Wang, Jin-ling Zhang, Shu-ping Chen, Yong-hui Gao, Jian-liang Zhang, Yu Chen, Yue Zhang, Pei-jing Rong, Jun-ling Liu

**Affiliations:** Department of Physiology, Institute of Acupuncture and Moxibustion, China Academy of Chinese Medical Sciences, Beijing, China

**Keywords:** glial cells, electroacupuncture, incisional neck pain, glutamate transporter-1, glutamate–aspartate transporter

## Abstract

**Objective:**

Glial cells are involved in the analgesic effect of electroacupuncture (EA) in rats with chronic neurological pain. The objective of this study was to observe the role of neuronal–glial interaction and glutamate (Glu) transporters in EA-induced acute neck pain relief in rats.

**Materials and methods:**

Male rats were placed into the following five groups: control, model, EA Futu (LI18), EA Hegu (LI4)-Neiguan (PC6), and EA Zusanli (ST36)-Yanglingquan (GB34). The incisional neck pain model was established by making a longitudinal incision along the midline of the neck. The thermal pain threshold (TPT) was measured using a radiation heat detector. The immunoactivities of glial fibrillary acidic protein (GFAP), ionized calcium-binding adapter molecule 1 (Iba-1), neurokinin-1 receptor (NK-1R), Glu aspartate transporter (GLAST), and Glu transporter-1 (GLT-1) in the dorsal horns (DHs) of the cervico-spinal cord (C2–C5) were detected using immunofluorescence histochemistry. The expression levels of GFAP, Iba-1, GLAST, and GLT-1 mRNAs were determined using quantitative real-time polymerase chain reaction (PCR).

**Results:**

The TPT and levels of mRNAs expression and immunoactivity of GLT-1 and GLAST were significantly decreased, and those of Iba-1 and GFAP were significantly increased in the model group than those of the control group (*P* < 0.05). The activated microgliacytes were gathered around the NK-1R positive neurons, and co-expression of NK-1R and astrocytes was observed in the model group. EA LI18 significantly increased the TPT and expression of GLAST and GLT-1 mRNAs (*P* < 0.05) and notably decreased the number of Iba-1 positive cells and Iba-l mRNA expression (*P* < 0.05), whereas GLAST and GLT-1 antagonists inhibited the analgesic effect of EA LI18. However, these effects, except for the downregulation of Iba-1 mRNA, were not observed in the EA ST36-GB34 group. Fewer NK-1R-positive neurons were visible in the spinal DHs in the EA LI18 group, and the co-expression of NK-1R and astrocytes was also lower than that in the three EA groups.

**Conclusion:**

Electroacupuncture of LI18 had an analgesic effect in rats with neck incisions, which may be related to its functions in suppressing the neuronal–glial cell interaction through NK-1R and upregulating the expression of GLAST and GLT-1 in the spinal DHs.

## Introduction

Postoperative incisional pain is a common complication of surgery that may seriously affect patient quality of life. Within 4 days after thyroidectomy or parathyroid and endoscopic surgery, many patients complain of severe neck pain and anterior chest discomfort (Wattier et al., 2016; Kim et al., 2018). Moreover, in some cases, chronic pain may occur due to a topical inflammatory reaction, central hypersensitivity, changes in patient psychological or pathophysiological factors, or improper treatment of acute pain (Lin, 2014; Lou et al., 2017). Block (2016) conducted a comprehensive analysis of previous studies and concluded that surgery-induced inflammation at the injury site further caused low-grade inflammation in the central nervous system (CNS), leading to an imbalance of neuronal–glial interaction and communication. Thus, enhanced excitability of the excitatory neurons and increased pain-signal transmission developed, inducing a transition from acute incisional pain to persistent postsurgical pain. During this process, the neuronal–glial interaction plays an important role in pain maintenance (Gwak et al., 2017; Zhang et al., 2017).

As excitatory neurotransmitters, glutamate (Glu) and substance P (SP) play a critical role in pain-signal transmission and maintenance. In the spinal dorsal horns (DHs), they are released from the peripheral endings of small diameter afferent fibers upon noxious stimulation (Kingery et al., 2002; Jin et al., 2009; Clarke et al., 2011), whereas, neurokinin-1 receptors (NK-1Rs) and Glu receptors (GluR) are expressed on spinal DH neurons (Ma et al., 2015; Wang et al., 2019). The NK-1Rs are also expressed in non-neuronal cells in the CNS, including microglia and astrocytes, and these glial cells have important immune functions (Härtel et al., 2009; Johnson et al., 2016; Burmeister et al., 2017; Jiang et al., 2020). The combination of SP and NK-1R functions in promoting inflammatory immune responses by the activated microglia and astrocytes (Johnson et al., 2016), which may be involved in acute incisional pain that progresses to chronic pain. The Glu aspartate transporter (GLAST) and Glu transporter-1 (GLT-1) are two major transporters expressed in astrocytes of the spinal cord that absorb extracellular Glu to maintain normal extracellular levels and protect neurons from neurotoxicity. Inhibition of GLAST expression in the spinal cord reduces excitatory synaptic activity and spontaneous responses after nociceptive stimulation of the paw (Niederberger et al., 2006). In the spinal DHs, GLT-1 and GLAST are densely expressed in laminae I and II, and their expression levels in the activated astrocytes (neither microglia nor neurons) were evidently decreased at both 7 and 14 days after partial sciatic nerve ligation (Xin et al., 2009). These results indicate that NK-1R, GLAST, and GLT-1 are all involved in the nociceptive input processing in DHs of the spinal cord.

Our previous research showed that EA of LI18 (close to the neck incision) and LI4-PC6 (located at the neighboring nerve segments of the neck incision) suppressed incisional neck pain or neck inflammatory pain and inhibited the immunoactivity of SP and NK-1R, as well as protein expression of the NR2B subunit of *N*-methyl-*D*-aspartate (NMDA) receptors in cervical spinal cord DHs in rats with inflammatory (Gao et al., 2009) and incisional neck pain (Qiao et al., 2010). Repeated EA treatment attenuated hyperalgesia by inhibiting spinal glial activation in rats with chronic neuropathic pain (Wang et al., 2018). Thus, EA may reduce pain by modulating SP/NK-1R signaling and GLAST activities, which have a close relationship with glial cells. However, it remains unclear whether the neuron–glial interaction *via* SP/NK-1R signaling and GLAST in the cervical spinal DHs are involved in EA-induced relief of acute incisional neck pain. Therefore, the present study was designed to investigate the role of spinal neuronal–glial cross-talk by regulating NK-1R and astrocytic GLAST in incisional pain induction and EA analgesia.

## Materials and methods

### Animals and grouping

All experimental protocols and animal care were approved by the Institutional Animal Welfare and Use Committee of the Institute of Acupuncture and Moxibustion of the China Academy of Chinese Medical Sciences (Approval No. 2013021801) and were performed in accordance with the National Institutes of Health Guide for the Care and Use of Laboratory Animals (NIH Publication No. 85–23, revised 1985). A total of 65 male Sprague-Dawley (SD) rats (200–220 g in weight) were obtained from the Experimental Animal Center of the China Academy of Medical Sciences [license number: SCXK (Jing, 2014-0013)] and housed under standard laboratory conditions at 22 ± 2^°^C with a 12:12-h light–dark cycle. The rats were given food and water freely and acclimatized to the laboratory conditions for 7 days prior to the experiment. Sixty-five rats were randomly assigned to the following five groups (*n* = 13 in each group): normal control, model (incisional neck pain), EA Futu (LI18), EA Hegu (LI4)-Neiguan (PC6), and EA Zusanli (ST36)-Yanglingquan (GB34) by using a random number table. To validate the effects of GLAST and GLT-1 in EA analgesia, another 30 male SD rats were randomized into the following three groups: vehicle + EA, glutamate aspartate transporter (GLIST) antagonist + EA, and GLT-1 antagonist + EA; these rats received intrathecal injection (i.t.) of the related vehicle or antagonists.

### Incisional neck pain model establishment

Rat neck hair was removed with an appropriate amount of hair removal cream (barium sulfide) 1 day before the incision operation. To establish the incisional neck pain model, the rats were anesthetized with isoflurane (1–2% in oxygen), delivered by a nose cone in a tabletop animal anesthesia ventilator system (VME Matrix, Midmark, Dayton, OH, USA). A 1.5 cm longitudinal incision was made along the midline of the neck, and a pair of forceps was used to separate and manipulate the bilateral sternohyoideus muscles in the region of the thyroid gland repeatedly for approximately 30 min. Next, the incision was sutured in layers with surgical silk (gauge 4.0) at intervals of approximately 0.5 cm. After the operation, the rats were placed back into the cages for recovery.

### Electroacupuncture intervention

**FIGURE 1:**
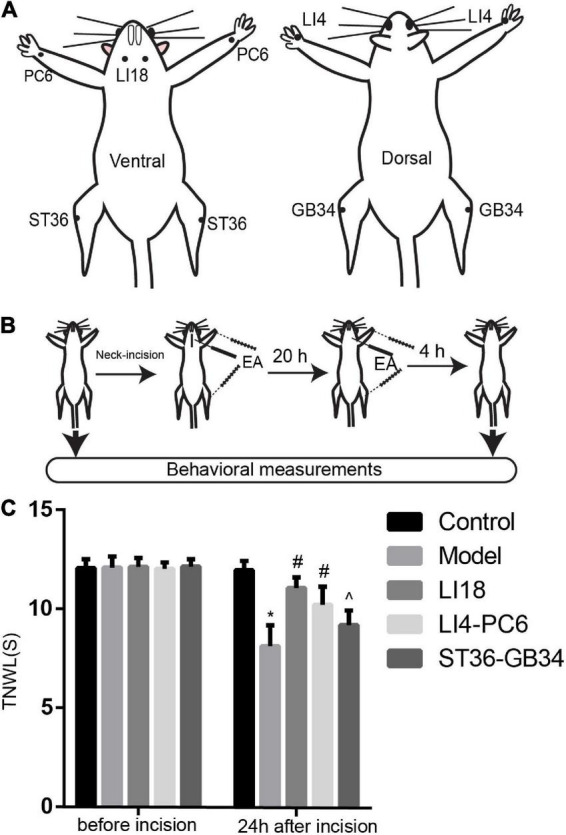
Electroacupuncture (EA) increases thermal pain threshold (TPT, thermal neck withdrawal latency, TNWL) in rats with incisional neck pain. **(A)** The location of LI18, LI4, PC6, ST36, and GB34 in rats. **(B)** Schematic diagram of experimental procedures of EA treatment and behavioral measurements. **(C)** Effect of EA at Futu (LI18), Hegu (LI14)-Neiguan (PC6), and Zusanli (ST36)–Yanglingquan (GB34) on TPT in rats with incisional neck pain (mean ± SD, *n* = 13/group). The TPT was significantly increased in both EA LI18 and EA PC6-LI4 groups but not in the EA ST36-GB34 group 24 h after incision. *P < 0.01, vs. the control group; #*P* < 0.05, vs. the model group; ^*P* < 0.05, vs. the EA LI18 group.

According to the acupoint chart for rats in the book “*Experimental Acupuncture*” (Li, 2007), and the positions of acupoints in the human body, LI18 is located on the lateral part of the neck between the anterior and posterior margins of the sternocleidomastoid muscle on the horizontal level of the 4th cervical vertebra. LI4 is located between the first and second metacarpal bones of the forelimb. PC6 is on the ventral side of the forelimb, approximately 3 mm from the wrist, between the ulna and the radius. ST36 is on the lateral side of the knee joint of the hind limb, 5 mm under the capitulum fibulae. GB34 is approximately 4 mm superior-lateral to ST36. Bilateral acupoints were selected for this study (Figure 1A).

Under the same anesthesia, isoflurane (1–2% in oxygen), the filiform needles were inserted into the acupoints mentioned above. EA treatment was administered using a HANS Apparatus (Hans-200A, Jisheng Medical Technology, Co., Ltd., Nanjing, China) immediately after the neck surgery was completed, and 20 h after the incision, with the following parameters: 1 mA, alternative frequency of 2/100 Hz, and duration of 30 min. The rats in the normal control and model groups were administered the same anesthesia without acupuncture needle insertion and EA stimulation.

### Measurement of thermal pain threshold

The thermal pain threshold (TPT, i.e., thermal neck withdrawal latency, TNWL) of the neck incision region was measured before and 24 h after neck incision using a tail-flick unit (37360, UGO Basile, Gemonio, VA, Italy) while the rat was fully awake (Figure 1B). The heat intensity was set to 50 units, with a cutoff time of 30 s to avoid tissue damage. The TPT was measured as described in our previous reports (Gao et al., 2009; Qiao et al., 2010). During the incisional neck pain measurement, the rat was held in place with the neck incision region over the mounted window of the radiant heat source of the tail-flick unit. The TPT was recorded automatically when the rat swiftly moved its neck away from the heat source. The measurement was repeated three times for each rat, with an interval of approximately 5 min between every two measurements, and the average value was used. The researcher who analyzed the TPT data was blinded to animal grouping and did not participate in EA interventions.

### Surgery for intrathecal injection

The rats used for intrathecal injection (i.t.) under light anesthesia (isoflurane) were placed in a stereotaxic apparatus. After exposing the dura mater of the lumbar (L5–L6) spinal cord, a polyethylene (PE10) catheter [outside diameter (OD) 0.61 mm, internal diameter (ID) 0.28 mm, Smiths Medical, ICU Medical Inc., Minneapolis, MN, USA] prefilled with sterilized 0.9% NaCl solution was inserted into the subdural space and moved rostrally about 8 cm to the spinal subarachnoid space of the cervical vertebrae C2–C5 (Chen et al., 2012). The local muscles and skin were sutured in layers with 3-0 silk stitches, the catheter was fixed and buried in the muscle layers and sealed with a cautery pen, and approximately 2–3 cm of the catheter end was left exposed. The rats were allowed to recover for 7 days before beginning the next experimental procedure. After completing the experiment, the location of the catheter was verified by injecting lidocaine, and only rats who developed a brief forelimb paresis after lidocaine injection were used.

### Intrathecal administration of glutamate aspartate transporter and glutamate transporter-1 antagonists

One week after catheter implantation, the rats were placed under light anesthesia (isoflurane) and received one of the following treatments by i.t.: 100 μg/10 μl DL-threo-β-benzyloxyaspartate (DL-TBOA, a competitive, non-transportable blocker of excitatory amino acid transporters such as GLAST), 100 μg/10 μl DHK (a GLT-1 specific antagonist), or 10 μl vehicle (0.9% saline, *n* = 10/group) through the catheter by using a micro-osmotic pump (0.5 μl/h), once daily for 5 days. Any residual reagent solution or vehicle was flushed from the catheter with subsequent delivery of 10 μl of saline, with the outer end sealed by heat every time. This treatment was followed by the neck incision procedure. Thermal hyperalgesia of the neck incision area was measured before incision, at 4 and 24 h after incision.

### Quantitative real-time polymerase chain reaction

Under deep anesthesia with pentobarbital sodium (35 mg/kg, i.p.), the C2–C5 segments of the cervical dorsal spinal cord (semi-section) were quickly collected on ice 24 h after neck incision and stored in liquid nitrogen. The total RNA of spinal cord tissue was extracted using the TRIzol method. The reverse transcription of cDNA was performed using the Prime ScriptTM Reagent Kit (Takara Bio, Shiga, Japan). Gene expression levels were measured using a fluorescence quantitative real-time PCR system (ABI7500, Applied Biosystems, Waltham, MA, USA), with the primer sequences summarized in Table 1. Each reaction mixture consisted of 2 μl cDNA, 10 μl REAL SYBR Mixture (2×), 0.8 μl (10 μmol/μl) of both forward and reverse primers, and 7.2 μl PCR-grade water, equating to a final volume of 20 μl. PCR was performed under the following conditions: 95^°^C, 30 s; 40 PCR cycles (95^°^C, 5 s, 60^°^C, 40 s); followed by 95^°^C for 10 s, 60^°^C for 60 s, and 95^°^C for 15 s. The data were analyzed by 2^–ΔΔCt^.

**TABLE 1:** Primer sequences.

Primers	Sequences	Length (bp)
Iba-1	Forward, 5′-AGCGAATGCTGGAGAAACTTG-3′ Reverse, 5′-AGTTGGCTTCTGGTGTTCTTTG-3′	194
GFAP	Forward, 5′-GCTAATGACTATCGCCGCCAACTG-3′ Reverse, 5′-CCTCCTGGTAACTCGCCGACTCC-3′	136
GLAST	Forward, 5′-GCCATCATGAGATTGGTAGCGGT-3′ Reverse, 5′-GGAAGTAGAGGAGAGGCAGGACGA-3′	187
Glt-1	Forward, 5′-GAACTTCGGTCAATGTAGTGGGCG-3′ Reverse, 5′-TGGACTGCGTCTTGGTCATTTCG-3′	132
GAPDH	Forward, 5′-TTCCTACCCCCAATGTATCCG-3′ Reverse, 5′-CCACCCTGTTGCTGTAGCCATA-3′	270

### Immunofluorescence labeling

Under deep anesthesia with pentobarbital sodium (35 mg/kg, i.p.), the rats were initially perfused with normal saline followed by a 4% paraformaldehyde solution. The cervical spinal cord (C2–C5, semi-section) was removed and dehydrated with 30% sucrose solution and then sliced into 40 μm thick sections with a freezing microtome (Thermo Fisher Scientific, Bremen, Germany). NK-1 and glial fibrillary acidic protein (GFAP)/ionized calcium-binding adapter molecule 1 (Iba-1), or GFAP and GLT-1/GLAST double immunofluorescence analyses were performed. Free-floating tissue sections were incubated in the following primary antibodies: rabbit anti-NK-1 (1:1,000, AB5060, Sigma-Aldrich, Burlington, MA, USA) and mouse anti-GFAP (1:1,000, 3670S, Cell Signaling Technology, Danvers, MA, USA)/goat anti-Iba-1 (1:500, ab5076, Abcam, Waltham, MA, USA), or mouse anti-GFAP and rabbit anti-GLAST (1:200, ab416, Abcam, Waltham, MA, USA)/rabbit anti-GLT-1 (1:200, ab41621, Abcam, Waltham, MA, USA) overnight at 4^°^C. Next, the sections were incubated with fluorescent secondary antibodies: Alexa Fluor 594-conjugated donkey anti-rabbit antibodies (1:500, A32754, Life Technologies, Waltham, MA, USA) and Alexa Fluor 488-conjugated donkey anti-mouse antibodies (1:500, A21202, Life Technologies, Waltham, MA, USA)/Alexa Fluor 488-conjugated donkey anti-goat antibodies (1:500, A11055, Life Technologies, Waltham, MA, USA), or Alexa Fluor 488-conjugated donkey anti-mouse antibodies (1:500, A21202, Life Technologies, Waltham, MA, USA), and Alexa Fluor 594-conjugated donkey anti-rabbit antibodies (1:500, A32754, Life Technologies, Waltham, MA, USA) for 2 h at room temperature. The 435/455 Blue Fluorescent Nissl (1:1,000, N21479, Invitrogen, Waltham, MA, USA) was also used for the identification of cellular nuclei. The images of three sections (within the superficial DHs) of each rat were captured with a fluorescent microscope (E600, Eclipse, Nikon,Tokyo, Japan) or a laser scanning confocal microscope (FV1200, Olympus, Tokyo, Japan) equipped with a digital camera (DP70, Olympus, Tokyo, Japan). Under the microscope, five areas in the same section of DHs of the spinal cord in each tissue section were randomly selected for counting the number of Iba-1 positive cells or for measuring the intensity of GFAP/GLAST/GLT-1 immunofluorescence with the Nikon Imaging Software (NIS) elements (Nikon, Tokyo, Japan). The mean number or the mean intensity of each section in the five areas was calculated. Next, the mean number or mean intensity of three slices from the same rat was taken as the positive cell count or fluorescence intensity of that rat. Control immunostaining was obtained by substituting the primary antibody with normal serum.

### Statistical analyses

All data were expressed as the mean ± standard deviation (mean ± SD) and were analyzed with IBM SPSS software (IBM Corp., Armonk, NY, USA). Repeated measures ANOVA was used to analyze the TPT data, and one-way ANOVA was used to analyze the remaining data from the present study. A *post-hoc* test for least significant difference (LSD) was performed to compare differences between the two groups. Statistical significance was set at *P* < 0.05.

## Results

### Effect of electroacupuncture on thermal pain

Before the neck incision, we found no significant differences in the TPT levels among the five groups (*P* > 0.05, Figure 1C). After neck incision, the TPTs were significantly decreased in the model group when compared with the control group at 24 h after neck incision (*P* < 0.05). Also, when compared with the model group, the TPTs were observably increased 24 h after neck incision in both the EA LI18 and EA LI14-PC6 groups (*P* < 0.05, Figure 1C), but not in the EA ST36-GB34 group (*P* > 0.05, Figure 1C). The TPT of the EA LI18 group was markedly higher than that of the EA ST36-GB34 24 h after neck incision (*P* < 0.05, Figure 1C).

### Effect of electroacupuncture on activities of microgliacytes and astrocytes

**FIGURE 2:**
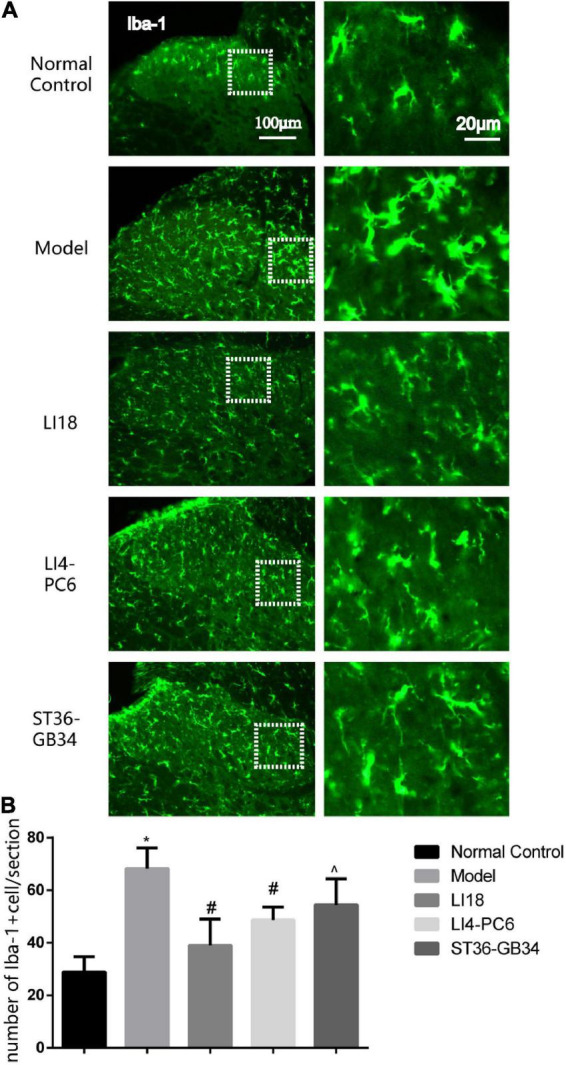
Comparison of the effects of electroacupuncture (EA) on activities of microglia in the dorsal horns (DHs) of cervical (C2–C5) spinal cord at 24 h after neck incision in different groups (mean ± SD, *n* = 5/group). **(A)** Representative fluorescence micrographs of immunofluorescent staining of ionized calcium-binding adapter molecule 1 (Iba-1) for microgliacytes in the spinal DHs in different groups. The magnified microglial cells shown in the right column images were chosen from the dashed squares of the left column images in the five groups. The scale bar in the five images on the left column is 100 μm, and 20 μm in the five images on the right column. **(B)** Bar graphs showing the numbers of microglia in the DHs of spinal cord at 24 h after incision in different groups. One-way ANOVA revealed that the number of Iba-1 immunoreaction (IR) positive cells was significantly increased in the model group (vs. the control group) and considerably decreased in both EA LI18 and EA PC6-LI4 groups but not in the EA ST36-GB34 group (vs. the model group). *P < 0.01, vs. the control group, #*P* < 0.05, vs. the model group, ^*P* < 0.05, vs. the EA LI18 group.

Under the microscope, 24 h after neck incision, the microgliacytes labeled by Iba-1 exhibited small cell bodies and thin processes in the superficial layers of the spinal DHs in the control group, and those in the model group exhibited an ameboid shape with enlarged cell bodies and thick and short processes (Figure 2A). When compared with the control group, the number of Iba-1 positive cells in the model group was observably increased 24 h after neck incision (*P* < 0.05, Figure 2B), suggesting microgliacyte activation after neck incision. The microgliacytes in the EA LI18 group exhibited a similar state as those in the control group. When compared with the model group, the number of Iba-1 positive cells at 24 h was significantly decreased in both the EA LI18 and EA LI4-PC6 groups (*P* < 0.05), whereas, the number in the EA ST36-GB34 was slightly decreased (*P* > 0.05). The number of Iba-1 positive cells was markedly lower in the EA LI18 group than that in the EA ST36-GB34 group 24 h after neck incision (*P* < 0.05, Figure 2B).

**FIGURE 3:**
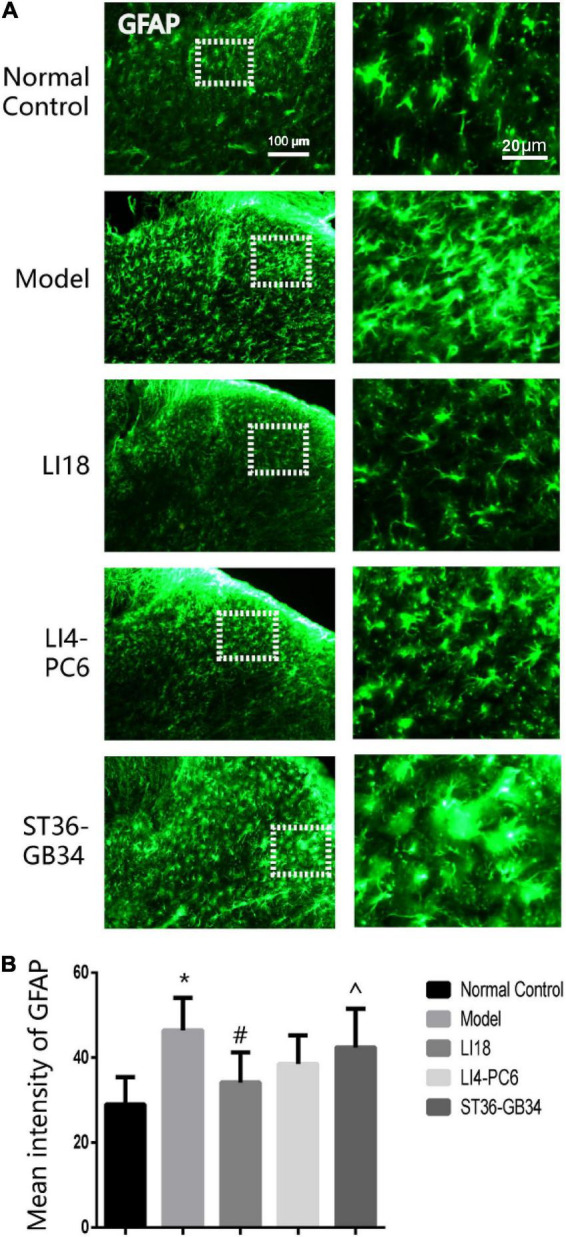
Effect of electroacupuncture (EA) on activities of astrocytes in dorsal horns (DHs) of cervical spinal cord at 24 h after neck incision in different groups. **(A)** Representative fluorescence micrographs of immunofluorescence staining of GFAP for astrocytes in the cervical (C2–C5) spinal DHs in the five groups. The magnified astrocytes shown in the right column images were chosen from the dashed squares of the right column images in the five groups. The scale bar in the five images on the left column is 100 μm, and 20 μm in the five images on the right column. **(B)** Bar graphs showing the mean intensity of glial fibrillary acidic protein (GFAP) immunoreaction (IR) in the DHs (mean ± SD, *n* = 5/group). One-way ANOVA analysis revealed that the mean intensity of GFAP IR was significantly increased in the model group (vs. the control group), and considerably decreased in the EA LI18 but not in the EA PC6-LI4 and EA ST36-GB34 groups (vs. the model group). *P < 0.01, vs. the control group, #*P* < 0.05, vs. the model group, ^*P* < 0.05, vs. the EA LI18 group.

In the normal control group, a few GFAP-labeled astrocytes were evenly distributed in the DHs of the cervical spinal cord and exhibited thin dendrites. The astrocytes in the model group exhibited an activated state marked by hypertrophied cell bodies and thicker processes, with a significantly increased mean intensity of GFAP than that in the control group (*P* < 0.05, Figure 3A). The average intensity of GFAP in the EA LI18 group (but not in the EA LI4-PC6 and ST36-GB34 groups, Figure 3B) was significantly lower than that in the model group 24 h after incision (*P* < 0.05), suggesting a suppression of the astrocytic activity only in the EA LI18 group after EA.

### Effect of electroacupuncture on the expression of ionized calcium-binding adapter molecule 1 and glial fibrillary acidic protein mRNAs

**FIGURE 4:**
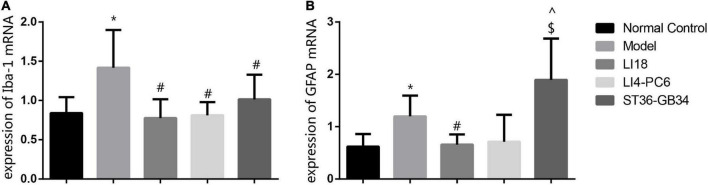
Effect of electroacupuncture (EA) intervention on ionized calcium-binding adapter molecule 1 (Iba-1) mRNA **(A)** and glial fibrillary acidic protein (GFAP) mRNA **(B)** of dorsal spinal cord (C2–C5) at 24 h after neck incision (mean ± SD, *n* = 8/group). The expression levels of Iba-1 mRNA and GFAP mRNA were significantly upregulated in the model group (vs. the control group), that of Iba-1 mRNA was obviously downregulated in the three EA groups (vs. the model group), and that of GFAP mRNA was obviously downregulated in the EA LI18 group but not in the EA PC6-LI4 and EA ST36-GB34 groups (vs. the model group). *P < 0.01, vs. the control group, #*P* < 0.05, vs. the model group, ^*P* < 0.05, vs. the EA LI18 group, $*P* < 0.05, vs. the EA LI14-PC6 group.

The expression levels of Iba-1 mRNA and GFAP mRNA in the cervical dorsal spinal cord in the model group were markedly higher than those in the normal control group 24 h after incision (*P* < 0.05, Figures 4A,B). When compared with the model group, the expression levels of Iba-1 mRNA and GFAP mRNA in the EA LI18 group, and those of Iba-1 mRNA in the EA LI14-PC6 and EA ST36-GB34 groups, were significantly decreased (*P* < 0.05, Figures 4A,B) 24 h after incision. Meanwhile, no remarkable differences were found among the three EA groups in the expression of Iba-1 mRNA, and between the EA LI18 and EA LI14-PC6 groups in the expression of GFAP mRNA. Also, no significant differences in the expression levels of GFAP mRNA were found between the EA ST36-GB34 group and the model group (*P* > 0.05, Figure 4B).

### Electroacupuncture suppresses the cross-talk between neurokinin-1 receptor and glial cells

To analyze the effect of EA on the interaction between NK-1R and glial cells in the cervical spinal cord, we examined the co-labeled state of NK-1R and Iba-1 or GFAP in the DHs by using an immunofluorescence dual-labeling technique. Confocal microscopic observation results indicated that the NK-1Rs expression was mainly on neurons but not on microgliacytes.

**FIGURE 5:**
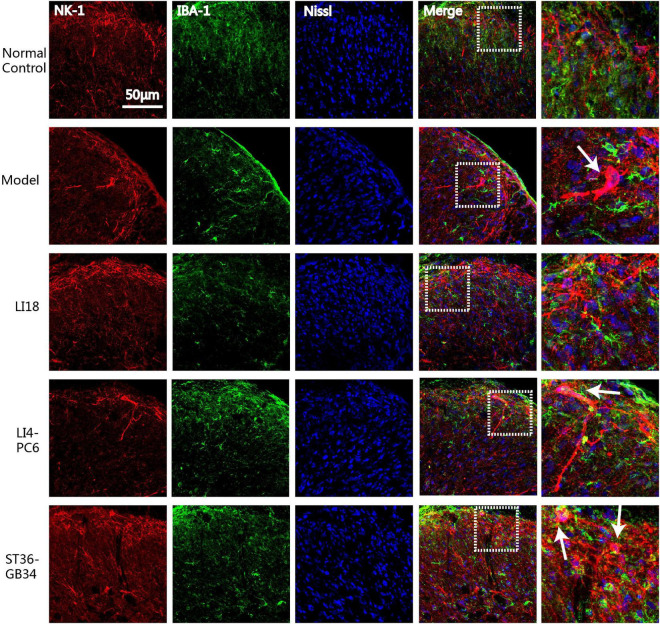
Confocal micrographs showing the expression of neurokinin-1 receptor (NK-1R) (red) and ionized calcium-binding adapter molecule 1 (Iba-1) (green) in the superficial dorsal horns (DHs) of the cervical spinal cord in different groups at 24 h after neck incision. Nissl staining (blue) shows the nucleus of cells. The scale bar is 50 μm. The white arrows indicate the NK-1 immunoreaction (IR) (red) positive neurons which are gathered around by the activated microgliacytes (green) in the DHs of cervical spinal cord.

In the normal control group, NK-1R expression was found in the superficial layers of the DHs of the spinal cord (lamina I) where both C and Aδ fibers terminate, and the Iba-1-labeled microglial cells remained in a resting state. NK-1R was clearly expressed on the neuronal bodies and axons (in the lamina I), which extended to the laminae II and III (Figure 5) in the model group 24 h after neck incision. Simultaneously, it was found that some activated microgliacytes gathered around the neurons expressing NK-1R in the spinal cord DHs. Following EA intervention, it was difficult to detect NK-1R-labeled neurons in the spinal cord DHs in the EA LI18 group, whereas NK-1R was expressed in neurons in the superficial layer of the spinal cord DHs in the EA LI4-PC6 and EA ST36-GB34 groups.

**FIGURE 6:**
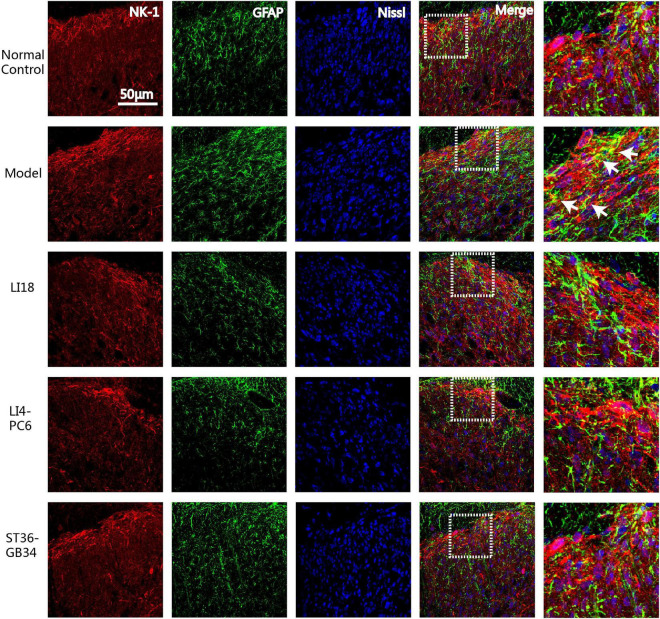
Confocal micrographs showing the co-expression (yellow) of NK-1 (red) and glial fibrillary acidic protein (GFAP) (green) in dorsal horns (DHs) of the cervical spinal cord in different groups at 24 h after neck incision. The scale bar is 50 μm. The white arrows indicate the NK-1R positive astrocytes. The expression of NK-1 immunoreaction (IR) and GFAP IR and co-expression were clearly seen in the superficial layer of spinal DHs (laminae II–III) in the model group and reduced in the three EA groups.

Immunofluorescence triple-labeling showed that few NK-1R- and GFAP-positive astrocytes were found in the superficial layers of spinal cord DHs in the normal control group rats (Figure 6), and many GFAP-labeled astrocytes were activated in laminae II–III 24 h after incision in the model group (Figure 6). This co-expression of NK-1R and GFAP was not found in the EA LI18 and EA LI4-PC6 groups, probably due to an EA-induced inhibitory effect on NK-1-positive astrocytes (Figure 6). Moreover, a markedly lower level of NK-1R-positive astrocytes was observed in the EA ST36-GB34 group.

### Effect of electroacupuncture on the expression of glutamate aspartate transporter and glutamate transporter-1

**FIGURE 7:**
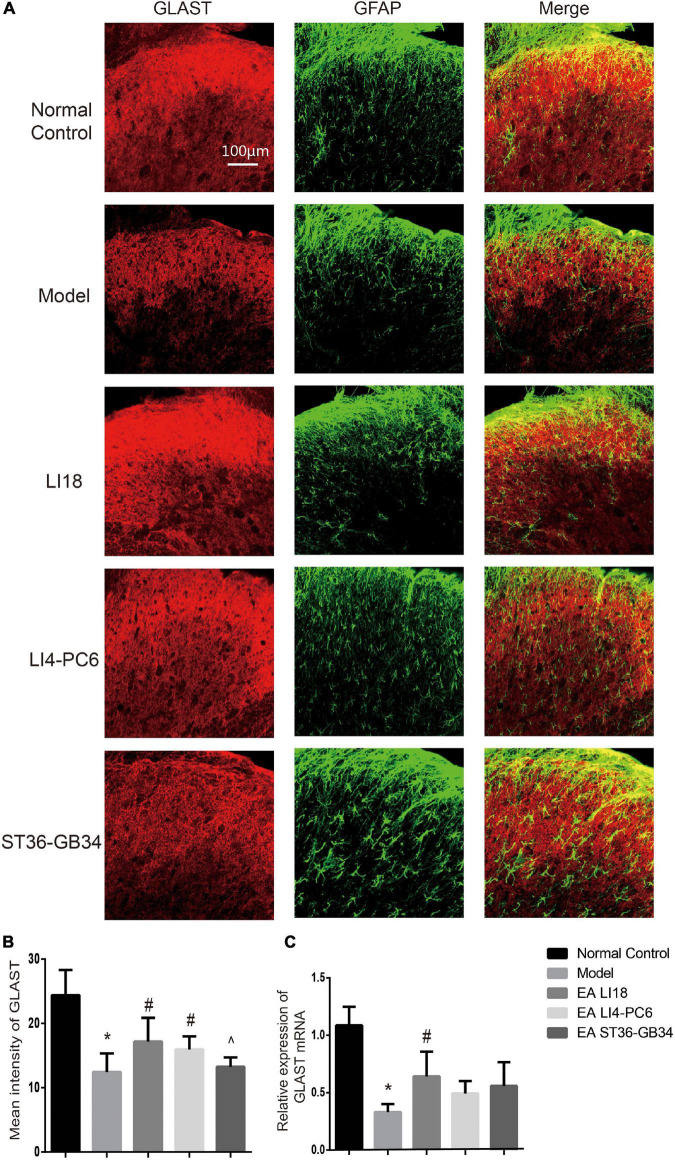
Comparison of expression of glu aspartate transporter (GLAST) in different groups (mean ± SD, *n* = 6/group). **(A)** Fluorescence micrographs of immunohistochemistry staining showing the co-expression (yellow) of GLAST (red) and glial fibrillary acidic protein (GFAP) (green) in the cervical spinal cord dorsal horns (DHs) at 24 h after neck incision. The scale bar is 100 μm. **(B)** Bar graphs showing the mean fluorescence intensity of GLAST in the five groups. The expression of GLAST protein was reduced in the model group relevant to the control group. After electroacupuncture (EA), the expression level of GLAST protein was significantly upregulated in both EA LI18 and EA PC6-LI4 groups but not in the EA ST36-GB32 group. **(C)** Bar graphs showing the relative expression of GLAST mRNA in the five groups. The expression of GLAST mRNA was significantly downregulated in the model group (vs. the control group) and significantly increased in EA LI18 group, rather than in the EA PC6-LI4 and EA ST36-GB34 groups (vs. the model group). *P < 0.01, vs. the control group, #*P* < 0.05, vs. the model group, ^*P* < 0.05, vs. the EA LI18 group.

**FIGURE 8:**
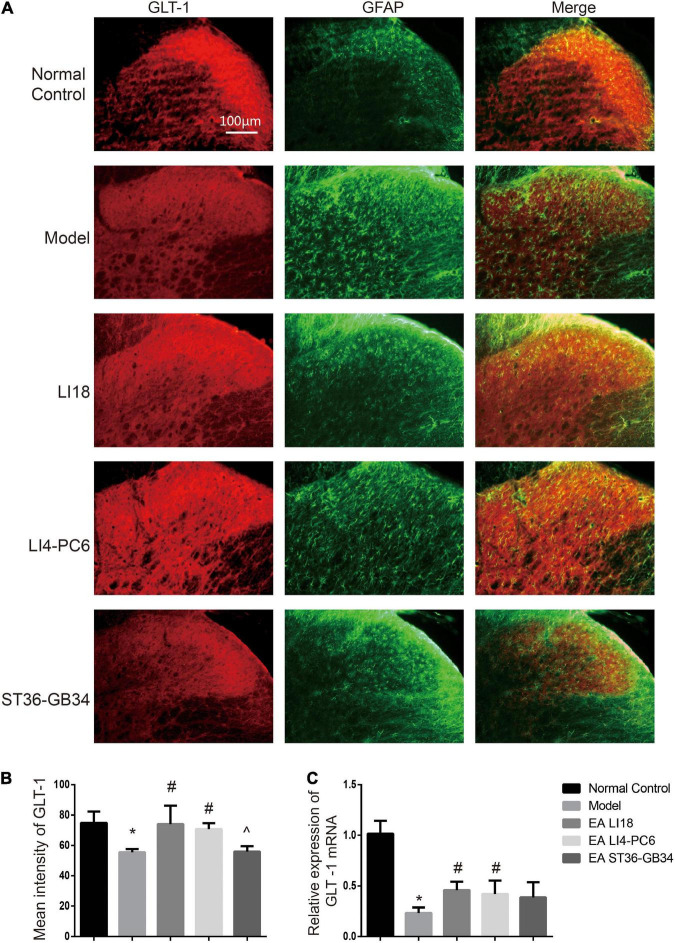
Comparison of expression of glu transporter-1 (GLT-1) in different groups (mean ± SD, *n* = 6/group). **(A)** Fluorescence micrographs of immunohistochemistry staining showing the co-expression (yellow) of GLT-1 (red) and glial fibrillary acidic protein (GFAP) (green) in different groups in the cervical spinal dorsal horns (DHs) 24 h after neck incision. GLT-1 was densely expressed by astrocytes in the normal control group, relatively denser in the EA LI18 and EA LI4-PC6 groups, and lower in both model and EA ST36-GB34 group. The scale bar is 100 μm. **(B)** Bar graphs showing the mean fluorescence intensity of GLT-1 in the five groups. GLT-1 expression was reduced in the model group relevant to the control group. After electroacupuncture (EA), the expression levels of GLT-1 were significantly upregulated in both EA LI18 and EA PC6-LI4 groups but not in the EA ST36-GB32 group (vs. the model group). **(C)** Bar graphs showing the relative expression of GLT-1 mRNA in the five groups. The expression of GLT-1 mRNA was observably decreased in the model group compared to the control group and significantly increased in both EA LI18 and EA PC6-LI4 groups (rather than in the EA ST36-GB34 group) in contrast to the model group. *P < 0.01, vs. the control group, #*P* < 0.05, vs. the model group, ^*P* < 0.05, vs. the EA LI18 group.

Immunofluorescence staining analysis showed that GLAST immunoreaction-positive product was found in the superficial layer of spinal cord DHs (lamina I) where C/Aδ fibers terminate and transmit nociceptive information (Figure 7A). When compared with the normal control group, the immunofluorescence intensity of GLAST and expression of GLAST mRNA were markedly decreased in the model group 24 h after neck incision (*P* < 0.05, Figures 7B,C). Moreover, when compared with the model group, the expression of GLAST mRNA in the EA LI18 group and the immunofluorescence intensity of GLAST in both the EA LI18 and EA LI4-PC6 groups were significantly increased (*P* < 0.05, Figures 7B,C), but this increase was not observed in the EA ST36-GB34 group (*P* > 0.05). When compared with the normal control group, the mean immunofluorescence intensity of GLT-1 and the expression of GLT-1 mRNA were significantly decreased in the model group (*P* < 0.05, Figures 8A–C). In contrast to the model group, the expression of GLT-1 immunofluorescence intensity and GLT-1 mRNA was significantly increased in both the EA LI18 and EA LI4-PC6 groups (*P* < 0.05, Figures 8B,C), but not in the EA ST36-GB34 group (*P* > 0.05). The effect of the EA LI18 group in upregulating the immunofluorescence intensity levels of GLT-1 and GLAST of spinal cord DHs was significantly superior to that of the EA ST36-GB34 group (*P* < 0.05, Figures 7B, 8B).

### Glutamate aspartate transporter and glutamate transporter-1 antagonists weaken the analgesic effect of electroacupuncture

**FIGURE 9:**
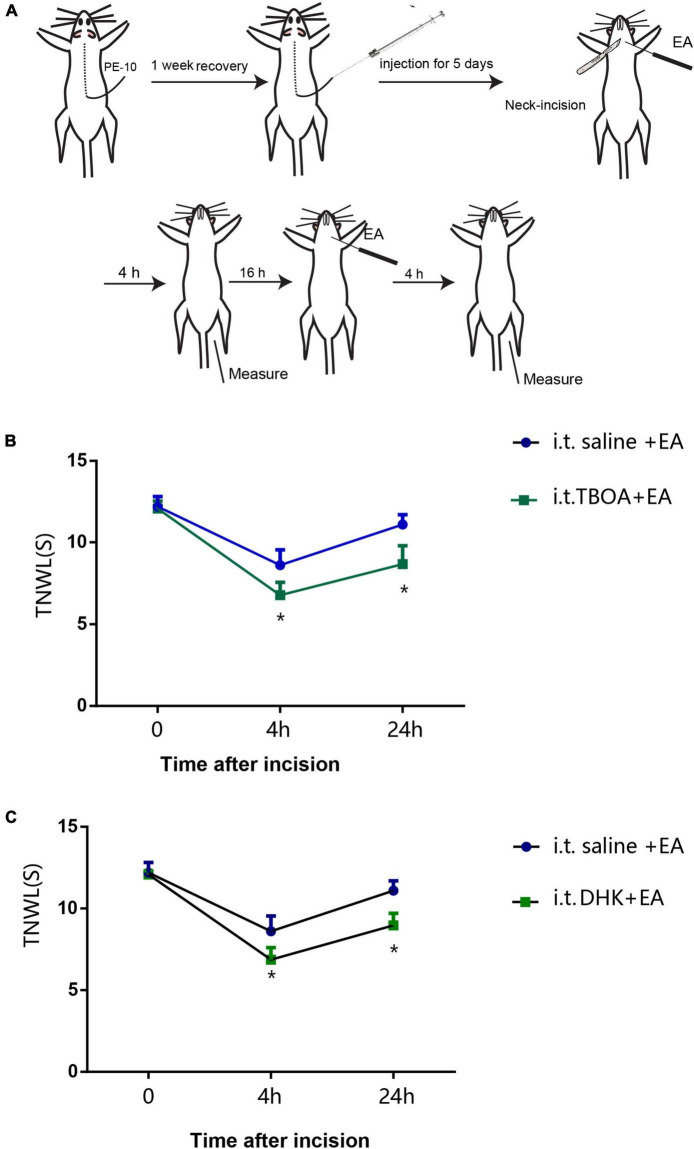
Intrathecal injection of glu aspartate transporter (GLAST) antagonist (DL-threo-beta-Benzyloxyaspartate, DL-TBOA) and glu transporter-1 (GLT-1) antagonist (dihydrokainate, DHK) diminishes the analgesic effect of EA LI18. **(A)** Schematic diagram showing the experimental procedures of i.t., electroacupuncture (EA) intervention of LI18, and behavioral measurements. **(B,C)** Significant reductions in the thermal pain threshold (TPT, thermal neck withdrawal latency, TNWL) at different time point after i.t. of GLAST antagonist TBOA **(B)**, and GLT-1 antagonist DHK **(C)**, separately (mean ± SD, *N* = 10 per group). i.t.: intrathecal injection; *P < 0.05, vs. the i.t.-saline + EA group.

To confirm the involvement of GLAST and GLT-1 in the analgesic effect of EA, we employed i.t. of antagonists of GLAST (TBOA) and GLT-1 (DHK) in rats with incisional neck pain (Figure 9A). The outcomes showed that following EA intervention of bilateral LI18, the TPT levels were significantly lower at 4 and 24 h after modeling in the i.t. TBOA + EA and DHK + EA groups than those in the i.t. saline + EA group (*P* < 0.05, Figures 9B,C). A marked weakened analgesic effect of EA was found after the administration of both GLAST and GLT-1 antagonists, suggesting that both GLAST and GLT-1 in cervical spinal cord DHs contribute to the analgesic effect of EA of LI18 in rats with neck incisions.

## Discussion

Our previous studies revealed that EA of LI18 and PC6-LI4 induced pain relief in rats with neck incision at 4 h after modeling (Qiao et al., 2010); however, the effect was not observed 24 h after neck incision when EA treatment was given twice. In the present study, an analgesic effect was observed after EA treatment was given twice in EA LI18 and EA LI4-PC6 groups (one immediately after the incision and another 20 h later) and was higher than that in EA ST36-GB34. These results were similar to the outcomes of Jia et al. (2016) in which EA of ST36 and Kunlun (BL 60) effectively reduced both mechanical and thermal hyperalgesia in rats with pelmatic incisional pain. The difference in the analgesic effect among the three acupoint groups is probably due to different segmental innervations. LI18 is close to the neck incision (in the same nerve segment, thus having the best EA effect), PC6 and LI4 are in the neighboring nerve segment, and ST38 and GB34 are quite distant from the neck incision, exhibiting a better and a poorer effect, respectively. Our previous electrophysiological research (Liu et al., 1993) showed that in normal rabbits, the spontaneous discharges of DH neurons in the thoracic spinal cord (T2–T3) were activated by EA stimulation of PC6 (the same nerve segment as T2–T3) in 35.48% (11/31) units and activated by EA of ST36 (the nerve segment distant from T2–T3) in only 7.14% (2/28) units. Moreover, acupuncture analgesia includes local analgesia that is achieved by activating A-type nerve fibers (innocuous intensity, triggering segmental nerve inhibition) and systemic analgesia which is obtained by activating Aδ and C nerve afferents (noxious stimulation). The latter may recruit the diffuse noxious inhibitory controls system (Xu et al., 2003; Zhu, 2015) of the brain stem. Thus, the stimulation strength is critical in achieving regional or systemic analgesic effects. These findings explain why in the present study, the analgesic effect of EA LI18 at 1 mA (lower strength) was significantly superior to that of EA ST36-GB34.

As the largest number of cells in the CNS, gliacytes play an important role in both physiological and pathological processes. Over the past several decades, a growing body of evidence indicated that activation of microglia and astrocytes was involved in pain induction and maintenance (Scholz and Woolf, 2007; Chen et al., 2018). The microgliacytes are essential for synaptic plasticity and chronic pain in the spinal cord DHs (Xu et al., 2014), and the activated astrocytes are involved in the pathogenesis of hypersensitivity in a chronic post-ischemia pain model (Fu et al., 2006). The findings of the present study showed that along with the appearance of incisional neck pain, the number of Iba-1-labeled microgliacytes was apparently increased and the GFAP-labeled astrocytes also exhibited an activation state, marked by hypertrophied cell bodies with thicker processes 24 h after neck incision. In addition, a notable upregulation of Iba-1 mRNA and GFAP mRNA was observed in the cervical spinal cord DHs 24 h after neck incision.

It is reported that even in the very early postoperative period after plantar incision, the peripheral sensory afferent neurons exhibited an increased spontaneous and stimulus-evoked activity that mediated hyperalgesia and allodynia (Xu and Brennan, 2009). Administration of SP produced mechanical allodynia in a dose-dependent fashion in mice, and administration of the NK-1R antagonist LY303870 attenuated the allodynia (Peyman et al., 2009). The SP is produced at high levels within the CNS, and NK-1R is abundantly expressed on neurons and is also present in glial cells including microglia and astrocytes (Johnson et al., 2016). C-fiber afferent SP/NK-1R signaling supports spinal microglial and astrocytic activation after noxious stimulation (Li et al., 2015). Thus, SP/NK-1 signaling plays an important role in nociceptive sensitization by facilitating the interaction of neuron–glial cells, and the relationship of NK-1R and glial cells during the occurrence of neck incision acute pain was observed by confocal imaging. After the neck incision, the increased NK-1R was expressed on the neuronal bodies in lamina I, and the activated microgliacytes gathered around the NK-1R-positive neurons. Co-expression of NK-1R and GFAP was observed in astrocytes in laminae II–III, which was deeper than those predominantly expressed in the NK-1R area. Therefore, NK-1R may be involved in the induction of hyperalgesia after neck incision through a glial-cell-mediated inflammatory response. Inhibiting a spinal glial cell activation and inflammation reaction may be one of the effective ways to mitigate pain. Our previous study showed that repeated EA suppressed the activated glial cells induced by chronic neuropathological pain (Wang et al., 2018), but the effect of EA on glial cells was unknown in rats with incisional pain. When EA intervention was applied twice in the present study, along with the occurrence of pain relief, the Iba-1 IR positive cells and expression of Iba-1 mRNA in both EA LI18 and EA PC6-LI4 groups, and the expression of GFAP mRNA in the EA LI18 group were significantly reduced. This indicate that suppression of activities of both microgliacytes and astrocytes may also contribute to the analgesic effect of EA in rats with acute pain. NK-1R is expressed by neurons and glial cells of the CNS. Our study demonstrated that in LI18 and LI4-PC6, EA stimulation-induced downregulation of SP and NK-1R immunoactivity levels in the dorsal cervico-spinal cord may have contributed to their effects in relieving neck incision pain (Qiao et al., 2010), but the relationship of the SP/NK-1R signal and the glial cells was not clear after EA treatment. The findings of the present study demonstrated that following EA intervention, almost no NK-1R-positive neurons were visible in the spinal cord DHs in the EA LI18 group, and the number of NK-1R-positive astrocytes was also fewer to be found in DHs of the 3 EA groups, suggesting that EA regulates the neuron–glial cell interaction through NK-1R to achieve an analgesic effect. These findings have not previously been demonstrated in similar incisional pain animal models.

Glutamate is a major excitatory neurotransmitter in the central neuronal circuits, including the spinal cord DHs, and plays an important role in the nociceptive sensory transmission from injured tissue peripherals to the DHs of the spinal cord (Neugebauer, 2007). When Glu is released from the presynaptic nerve endings, it acts on Glu receptors of the post-synaptic membrane, such as NMDA, a-amino-3-hydroxy-5-methyl-4-isoxazolepropionic acid (AMPA), and metabotropic Glu receptors. Activation of these receptors causes the spinal cord neurons to become more sensitive to peripheral inputs, leading to central sensitization. Excessive extracellular Glu induces an overload of Ca^2+^ influx that may result in excitotoxicity and neuronal death (Kung et al., 2013). The clearance of neurotoxic concentrations of Glu is completed by a high-affinity Glu uptake system made up of the following five types of Na^+^-dependent high-affinity Glu transporters: GLT-1, GLAST, excitatory amino acid carrier 1 (EAAC1), excitatory amino acid transporter 4 (EAAT4), and EAAT5. Among them, GLT-1 and GLAST are expressed mainly on glial cells and differentially expressed on sensory neurons and post-synaptic spinal interneurons (Gadea and Lopez-Colome, 2001). In addition to the major role in extracellular Glu removal, Glu transporters also have more sophisticated functions in the modulation of neurotransmissions, such as modifying the time course of synaptic events and the extent and pattern of activation and desensitization of receptors extending outside the synaptic cleft and at the neighboring synapses (Danbolt, 2001). The outcomes of the present study showed that after neck incision, the protein and mRNA levels of GLAST and GLT-1 were downregulated accordingly in the model group and significantly upregulated in both EA LI18 and LI4-PC6 groups (rather than in the EA ST36-GB34 group), along with the occurrence and relief of the incisional neck pain. GLAST and GLT-1 antagonists also weakened the analgesic effect of EA LI18. Immunofluorescence dual labeling revealed that both GLAST and GLT-1 were expressed in astrocytes of the spinal cord DHs. As in rats with incisional pain, EA treatment also significantly upregulated GLAST and GLT-1 in rats with neuropathic pain, and i.t. of glutamate transport (GT) inhibitors attenuated the EA-induced analgesic effect (Zeng et al., 2016). Cui et al. (2016) revealed that the expression levels of spinal GTs increased on days 2 and 4 and gradually decreased as the time of EA intervention increased. Our previous study showed that EA significantly downregulated the expression levels of mGluR5 mRNA and the NMDAR 2B subunit mRNA and protein in rats with neck pain after neck incision (Lin et al., 2012), or subcutaneous injection of formalin at the neck (Gao et al., 2009). Therefore, EA alleviates incisional acute pain possibly by decreasing the expression of mGluR5 and NMDAR 2B on neurons and increasing the expression of GLAST and GLT-1 to reduce the neurotoxicity of Glu.

This study had several limitations. First, SP/NK-1R and GLAST/GLT-1 are only part of the interaction mechanisms of EA on neurons and glial cells. Fewer results were obtained on interactions between the microgliacytes and astrocytes and between the neurons and microgliacytes. Second, the techniques used in the present study were relatively limited, reducing the depth of the research. Thus, further studies should be conducted to elucidate the underlying mechanisms of EA analgesia in the same incision pain model.

**FIGURE 10:**
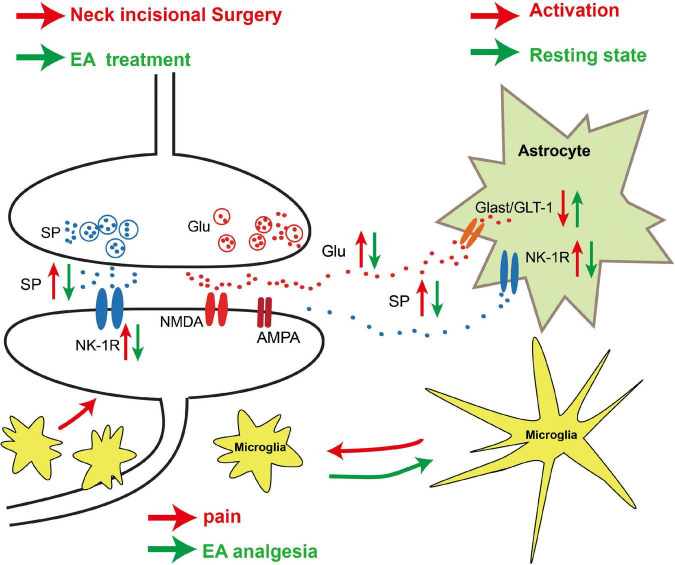
Schematic diagram showing the neuronal–glial mechanism of electroacupuncture (EA) analgesia in DHs of the cervical spinal cord in rats with incisional neck pain. Following neck incision, more substance P (SP) and glutamate (Glu) were released from the presynaptic terminals of the activated primary afferent nerve fibers in the superficial layers, and more neurokinin-1 receptor (NK-1R) was expressed on the post-synaptic membrane of the neurons and astrocytes. The expression levels of glu aspartate transporter (GLAST)/glu transporter-1 (GLT-1) on astrocytes were decreased, and more extracellular Glu acted on *N*-methyl-*D*-aspartate (NMDA)/amino-3-hydroxy-5-methyl-4-isoxazolepropionic acid (AMPA) of the post-synaptic neurons after neck incision. Those led to an activation of NK-1R-positive neurons and glial fibrillary acidic protein (GFAP)-labeled astrocytes and ionized calcium-binding adapter molecule 1 (Iba-1)-labeled microgliacytes. The activated microgliacytes gathered around the NK-1R-positive neurons. These activated neurons and glial cells may contribute to the formation of hyperalgesia. EA stimulation upregulated the expression of GLAST/GLT-1 on astrocytes and downregulated the expression of NK-1R on neurons and astrocytes, to reduce the extracellular Glu level and the transmission of injury information to the post-synaptic neurons. The microgliacytes and astrocytes returned to the resting state, and the aggregation of microgliacytes around the NK-1R-positive neurons was also alleviated after EA treatment. These changes between glial cells and neurons may be one of the mechanisms of EA analgesia.

In conclusion, our results demonstrate that EA of LI18 and LI4-PC6 can relieve thermal hyperalgesia probably by modulating neuronal–glial interaction in two ways, that is, by reducing SP/NK-1R signaling (nociceptive signal transmission) and glial cell activities, and by upregulating GLAST and GLT-1 to increase the intake of Glu in astrocytes in DHs of the cervical spinal cord (Figure 10). These results may provide experimental evidence for the clinical complementary application of EA of LI18 and LI4-PC6 in surgery of the thyroid gland region.

## Data availability statement

The original contributions presented in this study are included in the article/supplementary material, further inquiries can be directed to the corresponding authors.

## Ethics statement

The animal study was reviewed and approved by the Institutional Animal Welfare and Use Committee of the Institute of Acupuncture and Moxibustion of China Academy of Chinese Medical Sciences.

## Author contributions

J-LL, P-JR, and J-YW designed the experiments. J-LL and J-YW prepared the manuscript and figures. J-YW, J-LingZ, S-PC, YC, and YZ performed the experiments. Y-HG and J-LiangZ analyzed the data. All authors reviewed and approved the final manuscript.

## Abbreviations

EA: electroacupuncture
GLT-1: glutamate transporter-1
GLAST: glutamate–aspartate transporter
TPT: thermal pain threshold
Iba-1: ionized calcium-binding adapter molecule 1
GFAP: glial fibrillary acidic protein
CNS: central nervous system
NK-1R: neurokinin-1 receptor
GluR: glutamate receptors
SP: substance P
Glu: glutamate
LSD: least significant difference
NMDA: *N*-methyl -*D*-aspartate
EAAC1: excitatory amino acid carrier 1.
